# Reliability of breath by breath spirometry and relative flow-time indices for pulmonary function testing in horses

**DOI:** 10.1186/s12917-016-0893-3

**Published:** 2016-11-28

**Authors:** K. Burnheim, K. J. Hughes, D. L. Evans, S. L. Raidal

**Affiliations:** School of Animal and Veterinary Sciences, Charles Sturt University, Wagga Wagga, 2650 NSW Australia

**Keywords:** Lung function, Respiratory disease, Horse, Pneumotachography, Variability

## Abstract

**Background:**

Respiratory problems are common in horses, and are often diagnosed as a cause of poor athletic performance. Reliable, accurate and sensitive spirometric tests of airway function in resting horses would assist with the diagnosis of limitations to breathing and facilitate investigations of the effects of various treatments on breathing capacity. The evaluation of respiratory function in horses is challenging and suitable procedures are not widely available to equine practitioners. The determination of relative flow or flow-time measures is used in paediatric patients where compliance may limit conventional pulmonary function techniques. The aim of the current study was to characterise absolute and relative indices of respiratory function in healthy horses during eupnoea (tidal breathing) and carbon dioxide (CO_2_)-induced hyperpnoea (rebreathing) using a modified mask pneumotrachographic technique well suited to equine practice, and to evaluate the reliability of this technique over three consecutive days. Coefficients of variation, intra-class correlations, mean differences and 95% confidence intervals across all days of testing were established for each parameter.

**Results:**

The technique provided absolute measures of respiratory function (respiratory rate, tidal volume, peak inspiratory and expiratory flows, time to peak flow) consistent with previous studies and there was no significant effect of day on any measure of respiratory function. Variability of measurements was decreased during hyperpnea caused by rebreathing CO_2_, but a number of relative flow-time variables demonstrated good agreement during eupnoeic respiration.

**Conclusions:**

The technique was well tolerated by horses and study findings suggest the technique is suitable for evaluation of respiratory function in horses. The use of relative flow-time variables provided reproducible (consistent) results, suggesting the technique may be of use for repeated measures studies in horses during tidal breathing or rebreathing.

**Electronic supplementary material:**

The online version of this article (doi:10.1186/s12917-016-0893-3) contains supplementary material, which is available to authorized users.

## Background

Horses are affected by a number of discrete, non-infectious conditions causing reversible airway obstruction similar in many respects to asthma [1]. Recurrent airway obstruction (RAO) is a potentially debilitating condition affecting mature stabled horses in the northern hemisphere. Clinical signs of RAO are readily identified in affected horses, and include coughing, respiratory distress and increased respiratory effort [2]. Pulmonary function testing (PFT) has been used to characterise the condition, including acute exacerbations, and to demonstrate response to treatment. Inflammatory airway disease (IAD), a more subtle but very common condition worldwide, has been associated with poor performance, delayed respiratory recovery following exercise, and more severe exercise-induced hypoxaemia than in healthy, fit controls [3, 4]. As clinical signs of IAD may be subtle or inapparent in resting horses, recognition of the condition is more challenging and usually requires demonstration of altered bronchoalveolar lavage cytology, accumulation of tracheal exudate, and/or changes in respiratory function [1, 5].

Non-invasive PFT is the cornerstone of diagnosis for obstructive respiratory conditions in people, however PFT is challenging in horses due to high airflows, large pulmonary reserve and the inability of equine patients to follow instructions. Techniques adapted for evaluation of lung function in horses include measures of ‘conventional’ lung mechanics by simultaneous determination of transpulmonary pressure (estimated by oesophageal manometry) and airflow at the nostrils [6, 7], forced or impulse oscillation techniques [8, 9], flowmetric plethysmography [10] and forced expiratory techniques [11]. The ideal pulmonary function test is objective, repeatable, reproducible, accurate, valid, sensitive and specific [12]. In addition, in equine practice, the test would be non-invasive, acceptable to the horse and owner, and readily implemented in a field setting. Instruments should be robust and easily calibrated under field conditions.

Passage of an oesophageal pressure manometer may be unacceptable in a clinical setting, and conventional flow mechanics may lack sensitivity for the detection of IAD [13], although more recently the use of rebreathing techniques has improved the sensitivity of this technique [14]. Forced oscillation techniques require heavy sedation and specialised equipment ill-suited to a field setting. Open plethysmography, which uses inductance bands around the horse’s thorax and abdomen combined with penuemotachographic measurement airflow [10] is better suited to field conditions, and has been used to evaluate respiratory function in horses [5, 10, 15]. Forced expiration studies are common in human subjects, but in horses have been performed only in anaesthetised or heavily sedated patients [4, 11]. Increased tidal volumes and airflows have been elicited in conscious horses following exercise, by increased inspired CO_2_ concentration, or by administration of lobeline [14, 16–20].

In pediatric patients, where patient compliance is similarly limiting, relative flow indices have been developed to permit assessment of respiratory function at rest or during increased ventilatory manoeuvres. The ratio of time to reach peak expiratory flow to expiratory time (Tpef/Te) has been used to differentiate and characterise airflow obstruction in children and infants during forced expiratory breaths [21, 22] and tidal breathing [23]. As biphasic respiration, characteristic of healthy horses, is lost with obstructive conditions [24, 25], characterisation of relative flow values may prove helpful in equine patients. It has been proposed that relative flow-time relationships can indicate lower airway disease and negate the need to use maximal forced inspirations and expirations in horses [18], and the assessment of relative flow-time relationships has been used to demonstrate potential airflow limitations in horses with increased tracheal wash neutrophil percentages [26].

The current study was designed as a pilot study to characterise a novel pneumotachographic system for determination of absolute and relative flow-time indices of respiratory function in healthy horses during eupnoea (tidal breathing) and carbon dioxide induced hyperpnoea. In the context of PFT, Cotes et al. [12] have defined *repeatability* as the extent to which a result varies when it is repeated within a session, and *reproducibility* as the extent to which a result varies when it is repeated with changed condition, such as repeated measurements over a longer period of time. In the absence of an agreed ‘gold standard’ permitting assessment of accuracy, both concepts relate to measurement precision or reliability. Hence the aims of this study were to compare absolute indices of respiratory function derived using the mask pneumotachographic system with published results, and to determine the reliability of absolute and relative flow-time measures in healthy horses. We hypothesised that this approach would generate reproducible results in healthy, unsedated horses during eupnoeic breathing and hyperpnoea.

## Methods

### Horses

Eight healthy Standardbred mares, 3–13 years old (6.2 ± 3.3 years, mean ± standard deviation) and mean bodyweight 518 ± 29.8 kg, were used for this study. All horses were resident on site for at least 6 months prior to commencing the study, and none was routinely engaged in strenuous exercise. No abnormalities were evident in any horse following full clinical examination, including examination of body temperature, heart rate, respiratory rate and thoracic auscultation of left and right lung fields. Blood was collected for routine haematology and serum biochemistry to exclude systemic disease. For the duration of the study, no mare was coughing or had nasal discharge, and no horse had evidence of upper airway disease during endoscopic evaluation conducted subsequent to spirometry testing. Tracheal mucus was graded as previously described [27], and tracheal wash (TW) and broncholalveolar lavage (BAL) were performed at this time.

**Fig. 1:**
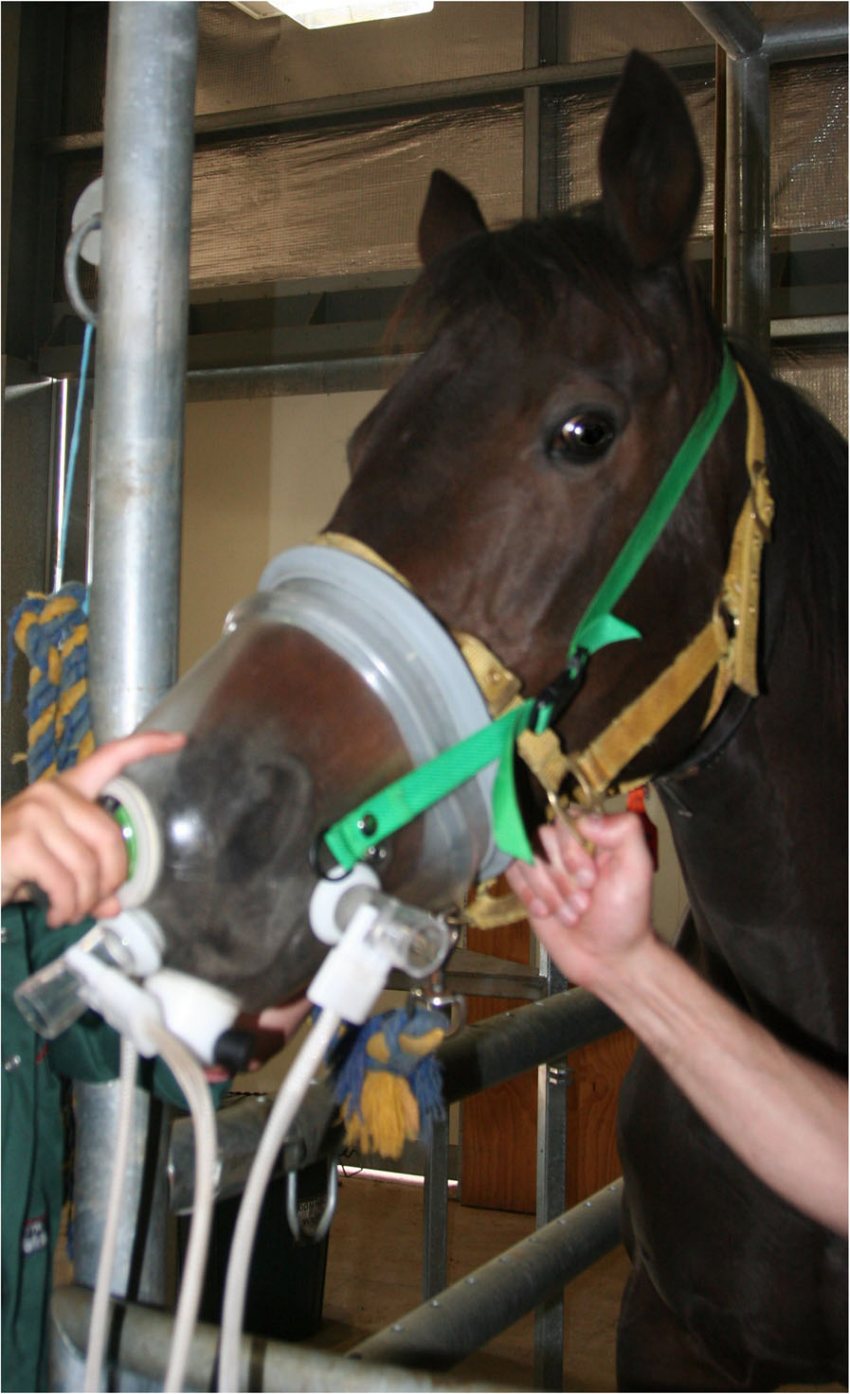
Facemask in position with three bi-directional pitot flow meters attached. Flow data was collected simultaneously from each sensor and summed to provide volume and flow results. Gas analysis was performed on samples aspirated continuously from the middle sensor. The horses head was maintained in the position shown throughout testing

Horses were accustomed to the stocks, testing area and a modified face mask (Aeromask, Trudell Medical International, distributed via Ranvet Banksmeadow NSW Australia) prior to commencement of the study. All pulmonary airflow analysis occurred between 11:00 and 15:00 h and horses were tested in the same order on experiment days, to reduce the effects of circadian rhythm on pulmonary function. An elevated head position was maintained throughout all spirometry procedures (Fig. 1). The daily temperature and relative humidity during measurements were, respectively, 16.7–20.3 °C and 41–76%.

The horses were kept in a one hectare paddock for the duration of the experiment with access to summer grass pasture, and were fed lucerne hay once daily. Horses were in a holding yard at for at least 30 min prior to testing to prevent increased respiratory rate due to exercise and excitement. Study design was approved by the Animal Care and Ethics Committee of Charles Sturt University, Wagga Wagga (ACEC approval number 10/077).

### Spirometry

A face mask (Aeromask, Trudell Medical International, distributed via Ranvet Banksmeadow NSW Australia) was modified to accommodate three bi-directional pitot flow meters (preVent Flow Sensor, MGC Diagnostics Corporation, St Paul, Minnesota) as described previously for breath-by-breath analysis of airflow [18, 28] and attached over the horse’s muzzle covering the nose and mouth (Fig. 1). Three pitot tubes were placed in 18 mm circular holes in the face mask: two tubes were positioned laterally and one was placed centrally. Each pitot sensor measured air flow to a maximum of 18 L/s, and the outputs from each tube were summed and recorded with a CPFS/D Laboratory Spirometer (MGC Diagnostics Corporation, St Paul, Minnesota). Gas flow was measured to 0.1 L/s resolution every 10 milliseconds, and tubes were calibrated using a seven litre certified calibration syringe (Hans Rudolph Incorporated, Shawnee, Kansas) prior to pulmonary function testing. Calibration was successful if the total flow in the tubes differed from the reference by less than two percent (range 0.20–1.90%, mean 0.74%). Flow rates were calibrated to body temperature and pressure, saturated with water vapour (BTPS) based on daily measurement of barometric pressure, ambient temperature and relative humidity. Oxygen and carbon dioxide concentrations were determined (ML206 Gas Analyser, ADInstruments, Bella Vista, NSW) in expired gas via a sensor incorporated into the central flow tube and following a two-step calibration procedure utilising room air and Carbogen (5% carbon dioxide, 95% oxygen; Carbogen, BOC, North Ryde, NSW). A rubber seal and stoppers were used to ensure the face mask was airtight and mask fit was assessed visually before and during every recording to ensure it was airtight.

Spirometry studies were conducted over consecutive days. Temperature, relative humidity and ambient pressure were recorded immediately prior to each spirometry test. Horses were loaded into familiar stocks and fitted with the face mask. Once settled, the three pitot tubes (each with umbilicus attached) were connected to the mask and five minutes of eupnoeic breathing was recorded. After completing spirometry on horses during eupnoeic breathing, a plastic bag (capacity 60 L) was placed over the mask and pitot tubes. The bag was held in place by one or two assistants who ensured an airtight seal was maintained between the bag and mask and that the bag was not interfering with airflow through the pitot tubes. Recording started once the bag was in place and rebreathing continued until carbon dioxide levels reached a plateau or exceeded 4.5% end tidal CO_2_, tidal volume was greater than 15 L or the horse no longer tolerated the rebreathing bag (typically two to three minutes, mean 4.8 ± 0.54% end tidal CO_2_). The bag was then removed and recording of spirometry measurements was continued until the horse’s tidal volume and flow measurements returned to resting values, at which time the mask was removed.

### Spirometry measurements

Spirometry recordings were evaluated from 5 min of eupnoeic breathing and from the final 2 to 3 min of carbon dioxide induced hyperpnoeic respiration. Three breaths were chosen for analysis from each recording that were free of artefacts such as swallowing or head movement, evidenced less than 10% difference in inspiratory and expiratory volume, and had the same flow curve with peak inspiratory and expiratory flow at similar locations for the three breaths (that is, all breaths evidenced early or late flow peaks and were considered representative of tidal breathing throughout the trace) (Additional file 1: Figure S1). Representative breaths during rebreathing were selected from the final 30 s of rebreathing when respiratory excursions were greatest. Where possible, three consecutive breaths were selected, however as ventilation during both eupnoea and rebreathing was labile, analysis of consecutive breaths was not always possible.

**Fig 2:**
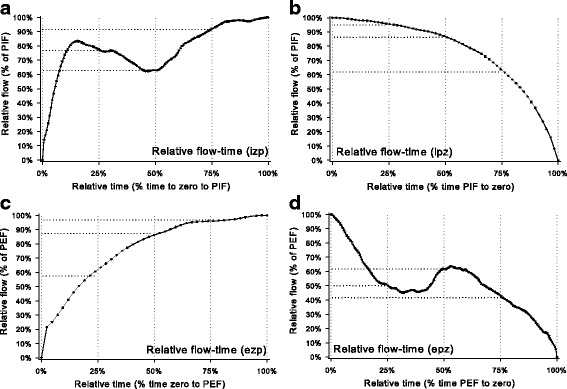
Relative flow-time graphs during a representative breath. Airflow is plotted as a percentage of peak inspiratory (**a** and **b**) or expiratory (**c** and **d**) flow. Figures **a** and **c** show flow between zero and peak, Figures **b** and **d** show flow from peak to zero. Relative flows are shown at 25, 50, and 75% of each phase of the respiratory cycle. Biphasic respiration is evident in Figures **a** and **d**

Respiratory frequency (Rf), tidal volume (V_T_), total breath period (Tt), inspiratory and expiratory periods (Ti and Te), peak inspiratory and expiratory flows (PIF and PEF), time to PIF (Tpif) and time to PEF (Tpef) were measured for each selected breath. Minute ventilation (MVe), and ratios of Te to Tt, Te to Ti, Tpef to Te and Tpif to Ti were calculated for each selected breath (Additional file 1: Table S1). Relative flow-time variables were calculated as described by [18] for each breath to determine the percentage of peak expiratory flow (PEF) at 25, 50 and 75% of the time from commencement of expiration to peak expiratory flow (Tpef) and were identified as ezp_25%_, ezp_50%_ and ezp_75%_, respectively. Similar relative flow-time measurements were made for 25, 50 and 75% of the time from Tpef to end of expiration (epz_25%_, epz_50%_ and epz_75%_). Analogous zero to peak (zp_%_) and peak to zero (pz_%_) measurements were determined for inspiratory phases of each breath (Fig. 2). Definitions of all relative flow-time indices are provided in Additional file 1: Table S2.

### Statistical methods

The repeatability and reproducibility of PFT methodology was assessed during eupnoeic breathing and rebreathing induced hyperpnoea by comparison of results for six indices of respiratory function (Rf, Vt, MVe, PEF, PIF, Tpef), two relative flow measures (Tpef/Te and Te/Tt) and 12 relative flow values (izp25%, izp50%, izp75%, ipz25%, ipz50%, ipz75%, ezp25%, ezp50%, ezp75%, epz25%, epz50%, epz75%) on each of the three days tested. Repeatability was assessed by determination of the coefficient of variation (CV) for the three breaths selected for analysis on each day. Reproducibility was assessed by comparison of mean results (average of three selected breaths) obtained on each of three days. Descriptive data is reported as the mean ± standard deviation for each the three days, and the CV across all days. The effect of repeated sampling (day) on indices of respiratory function was compared by one-way repeated measures analysis of variance, with separate analyses for eupneoic and hyperpnoeic ventilation. Data were checked for normality, equal variance and sphericity (equal variability of differences), and the Geisser-Greenhouse correction was applied as required. Where a significant replicate (day) or individual horse effect was observed, multiple pair-wise comparisons were performed using the Tukey test. Attempts were made to transform data that did not conform to a normal distribution or, if unsuccessful, such data was analysed by non-parametric analysis (Friedman one-way RM-ANOVA on ranks with post-hoc testing using Dunn’s multiple comparisons test). Intra-class correlation co-efficients (ICC) were calculated [29] for each measurement on each day tested using the F statistic derived from analysis of variance [30], with the 90% confidence interval calculated as previously described [31].

Possible associations between tracheal secretions and airway cytology were evaluated by graphing the endoscopic grade for tracheal secretions, percentage of neutrophils in tracheal wash cytology (TW % PMN), and the percentage of neutrophils (BAL % PMN), mast cells (BAL % mast cells) and eosinophils (BAL % eosinophils) in BAL fluid against measured or derived indices of pulmonary function. In addition, results were compared for horses that exhibited endoscopic (grade 2 tracheal secretions) or cytological evidence (BAL % PMN >10%, BAL % mast cells >5% or BAL % eosinophils >5%) of IAD [1] (*n* = 4) against the remaining horses with normal endoscopic and airway cytology findings. Spearman or Pearson correlations were calculated for categorical or continuous data (respectively) where a possible effect was evident graphically. All analyses were performed using GraphPad Prism version 6.0 for Windows (GraphPad Software, San Diego, California, USA, www.graphpad.com).

## Results

Endoscopic evaluation of the upper airway was unremarkable for each horse. Tracheal mucus scores and cytological results of TW and BAL samples are shown in Table 1. Whilst no horse demonstrated clinical signs suggestive of respiratory disease, five of the eight horses had increased tracheal secretions (grade 2), increased neutrophils in tracheal wash cytology, or fulfilled cytological definitions of IAD (increased BAL neutrophils, mast cells or eosinophils) [1]. The mask and rebreathing apparatus were tolerated well by unsedated horses, although considerable time was invested prior to commencing the study to acclimatise horses to PFT procedures. Ambient temperature (20.2 °C, 19.6 °C, 20.3 °C), humidity (58%, 52%, 76%) and atmospheric pressure (991 millibars, 996 millibars, 960 millibars) varied slightly on the three days of testing untreated horses.

**Table 1:**
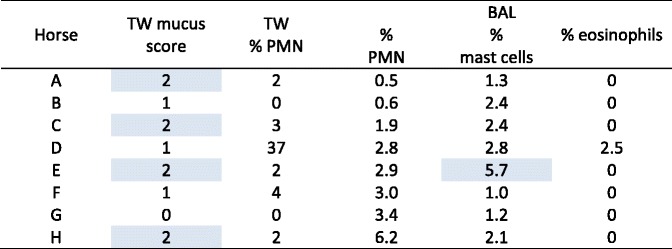
Endoscopic tracheal mucus score and cytology results for study horses. Results that fulfil endoscopic or cytological definitions for inflammatory airway disease [1] have been highlighted

**Fig. 3:**
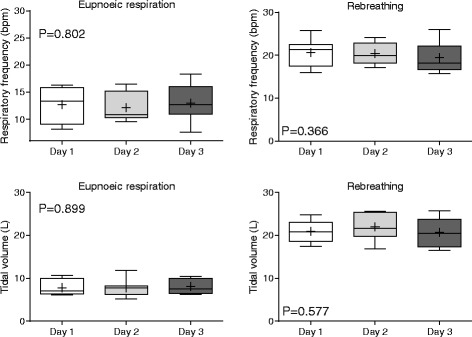
Respiratory frequency and tidal volume determination on each day of testing (reproducibility). Results are shown as mean (*cross*), median (*horizontal line*), quartiles (*box*) and 10–90th percentile (whiskers). Results from each day of testing represent the mean of three breaths analysed for each horse. P values were determined by one-way RM-ANOVA

**Fig. 4:**
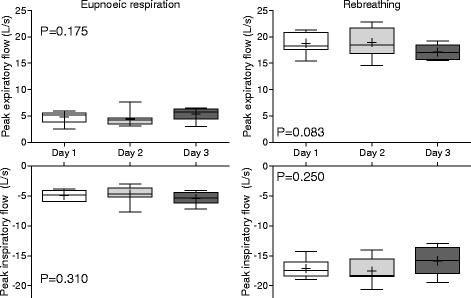
Peak inspiratory and expiratory flows on each day of testing (reproducibility). Results are shown as mean (*cross*), median (*horizontal line*), quartiles (*box*) and 10-90th percentile (whiskers). Results from each day of testing represent the mean of three breaths analysed for each horse. P values were determined by one-way RM-ANOVA

Absolute and relative measurements determined from data obtained during spirometry testing with the modified face mask are shown in Table 2. Respiratory frequency, tidal volume and peak flows are shown in Figs. 3 and 4; differences in means for each pair of tests (Day 1 vs Day 2, Day 2 vs Day 3, Day 1 vs Day 3) are provided (Additional file 1: Figure S2). Repeatability (comparison of the three selected breaths on the same day of testing) was high for most parameters, as within day CV’s were less than 10% for all parameters except time to peak expiratory and inspiratory flows. Rebreathing was associated with reduced variation relative to eupnoeic breathing, evidenced by lower within day and between day CVs. During eupnoeic breathing the position of both inspiratory and expiratory peak flow was highly variable for each horse, with peak flow occurring variably and seemingly randomly in early, middle or late inspiration and expiration for all horses. Biphasic respiration was noted during both inspiration and expiration, resulting in non-uniform changes in flow during each stage of the respiratory cycle in some horses. Rebreathing was associated with marked increases in respiratory frequency, airflow, tidal and minute volumes. Whereas PIF was higher than PEF during eupnoeic breathing, rebreathing was associated with higher PEF than PIF, and both PIF and PEF tended to occur closer to the start of inspiration or expiration, evidenced by lower Tpif and Tpef values.

**Table 2:**
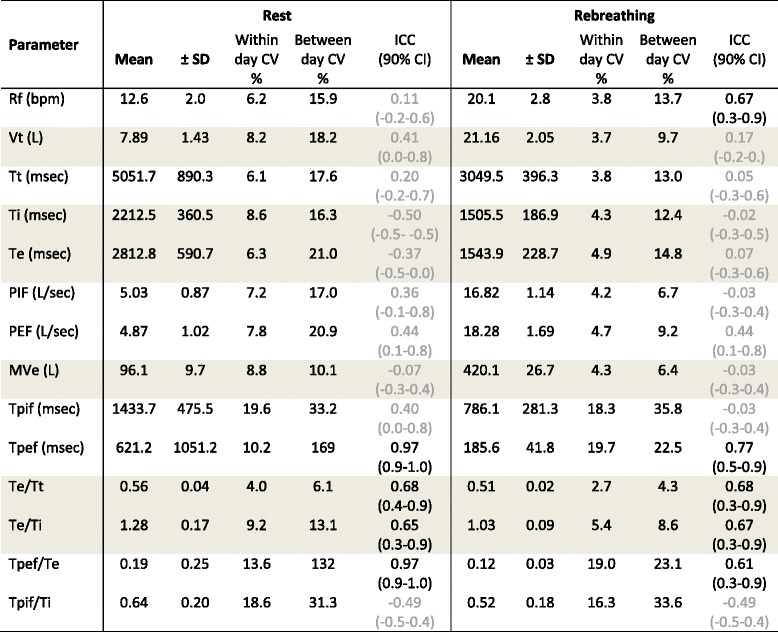
Mean ± standard deviation (SD) for absolute and relative spirometry measurements from eight untreated horses at rest (eupnoea) and during rebreathing over three consecutive days

Each parameter was determined by analysis of three breaths on each testing day, and within day coefficients of variation (CV) were calculated across this data as a measure of repeatability (within day variation). Mean values (± SD) reported below, between day CV (reproducibility), and intraclass correlation coefficients (ICC, reported with 90% confidence interval), were determined by analysis of pooled data (mean of three breaths) collected on each day of testing. Ghosted (grey) results for ICC indicate poor or moderate agreement

*Abbreviations: Rf* respiratory frequency, *bpm* breaths per minute, *Vt* tidal volume, *L* litres, *Tt* total breath period, *msec* milliseconds, *Ti* inspiratory period, *Te* expiratory period, *PIF* peak inspiratory flow, *PEF* peak expiratory flow, *L/sec* litres per second, *MVe* minute ventilation, *Tpif* time to PIF, *Tpef* time to PEF

A significant day effect was observed for the relative flow value Tpef/Te during eupnoeic breathing (*P* = 0.010, determined by Friedman one-way RM-ANOVA on ranks). No significant differences attributable to day of testing were observed for any other parameter. Higher CVs were evident when comparing results across the three days tested, by comparison with repeatability analyses on each day, indicating that variability was greater between days than within traces obtained on a single day. Results calculated for ICC values were consistent with strong agreement across days for Rf (during rebreathing only), and for Tpef, Te/Tt, Te/Ti and Tpef/Te (Table 2), suggesting that values relating to the time of PEF were consistent for individual animals across the three days. Significant differences were evident between individual horses for PEF during eupnoeic respiration and rebreathing (*P* = 0.025 and *P* = 0.026, respectively). Mean differences for relative flow variables are shown in Additional file 1: Figure S3.

**Fig. 5:**
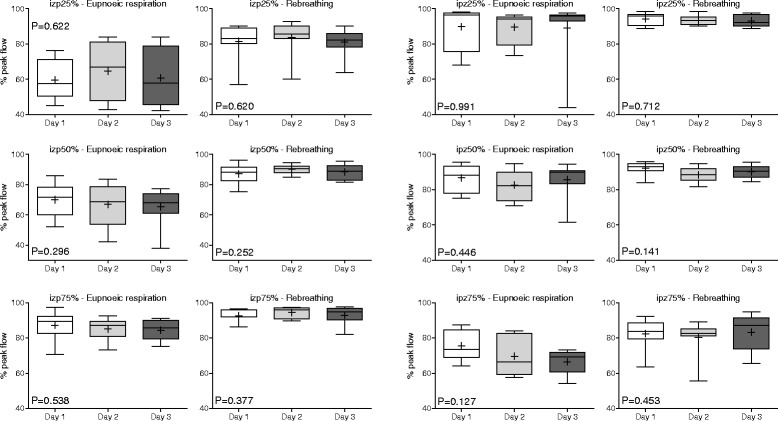
Inspiratory relative flow-time values on each day of testing (reproducibility). Results are shown as mean (*cross*), median (*horizontal line*), quartiles (*box*) and 10–90th percentile (whiskers). Results from each day of testing represent the mean of three breaths analysed for each horse. P values were determined by one-way RM-ANOVA. Inspiratory zero to peak (izp) values represent the relative percentage of peak inspiratory flow measured at 25, 50 and 75% of the time from zero to peak flow; inspiratory peak to zero (ipz) values represent the relative percentage of peak inspiratory flow measured at 25, 50 and 75% of the time from peak to zero flow

**Fig. 6:**
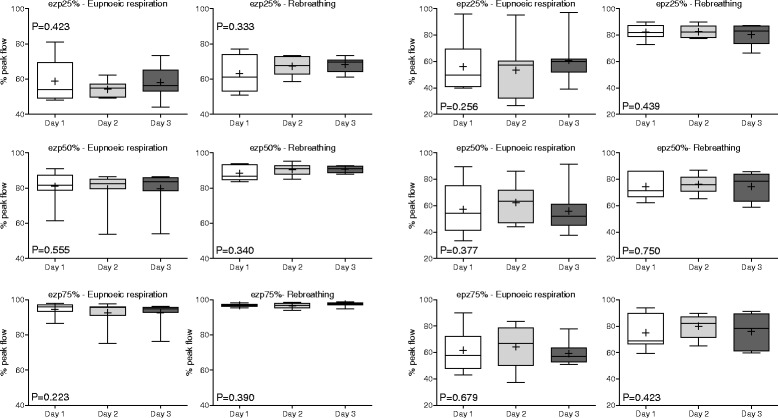
Expiratory relative flow-time values on each day of testing (reproducibility). Results are shown as mean (*cross*), median (*horizontal line*), quartiles (*box*) and 10-90th percentile (whiskers). Results from each day of testing represent the mean of three breaths analysed for each horse. P values were determined by one-way RM-ANOVA. Expiratory zero to peak (ezp) values represent the relative percentage of peak inspiratory flow measured at 25, 50 and 75% of the time from zero to peak flow; expiratory peak to zero (epz) values represent the relative percentage of peak inspiratory flow measured at 25, 50 and 75% of the time from peak to zero flow

Relative flow-time variables (Table 3) did not demonstrate any significant effect attributable to the day on which they were determined. Inspiratory and expiratory relative flow-time variables are shown in Figs. 5 and 6, respectively; differences in means for each pair of tests (Day 1 vs Day 2, Day 2 vs Day 3, Day 1 vs Day 3) are provided (Additional file 1: Figure S4). Repeatability (comparison of the three selected breaths on the same day of testing) and reproducibility (comparison of results across three days) were more variable during eupneoic respiration than was observed for absolute and relative flow values. Values obtained during rebreathing evidenced substantially less variation, but significant differences were observed between individual horses for all relative flow-time variables during eupnoeic respiration and/or rebreathing. Agreement was strongest, indicated by high ICC values, for expiratory flows around PEF (ezp50%, ezp75% and epz25%) during eupnoeic respiration (Table 3).

**Table 3:**
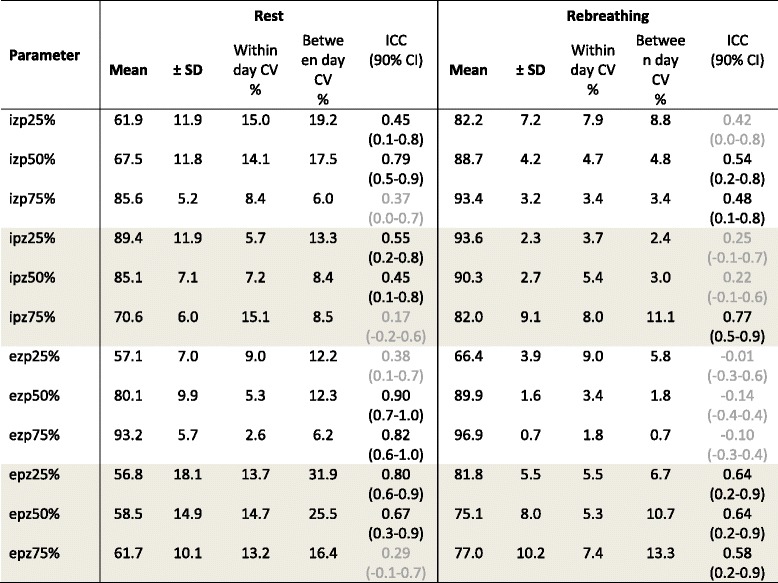
Mean ± standard deviation (SD) for relative flow measurements from eight untreated horses at rest (eupnoea) and during rebreathing over three consecutive days

Each parameter represented the percent of peak flow at the appropriate relative time within each breath cycle, and was determined by analysis of three breaths on each testing day. Within day coefficients of variation (CV) were calculated across this data as a measure of repeatability. Mean values (± SD) reported below, between day CV (reproducibility), and intraclass correlation coefficients (ICC, reported with 90% confidence interval), were determined by analysis of pooled data (mean of three breaths) collected on each day of testing. Ghosted (grey) results for ICC indicate poor or moderate agreement

*Abbreviations: izp* inspiratory flow zero to peak, *ipz* inspiratory flow peak to zero, expiratory flow zero to peak, *epz* expiratory flow peak to zero, *SD* standard deviation, *ICC* intraclass correlation coefficient, *CI* confidence interval

Consistent with the equivocal endoscopic and cytological findings from healthy horses in the current study, comparison of absolute and relative flow-time indices against endoscopic and cytological definitions of IAD demonstrated no significant associations between airway cytology and spirometry results in these horses.

## Discussion

In the current study, horses averaged a tidal volume of 7.89 ± 1.43 L (mean ± stdev) and respiratory frequency of 12 bpm during eupnoeic respiration, which is similar to previous studies using ultrasound or Fleisch-type pneumotachometers [32–35], and slightly greater than values determined by respiratory inductance plethysmography and pneumotachography [25, 36]. In addition to differences due to technique, it is possible that equipment differences, such as mask volume and airflow resistance, or physiological differences between horses, such as age [36], use [35] or sub-clinical airway disease [34, 35], may influence values determined during pulmonary function testing. Hyperpnoea induced by carbon dioxide rebreathing was investigated in the current study because previous studies have suggested that PFT during tidal breathing is insensitive for detection of subtle changes, such as those associated with IAD [14, 16, 17]. Bag rebreathing as a respiratory stimulus in the current study resulted in a 1.6 times increase in respiratory frequency and 2.8 times increase in tidal volume compared to eupnoeic breathing. Tidal volumes were consistent with previous results derived from hypercapnia [14, 16], and slightly greater than results reported during or following exercise [16, 18, 20]. Values for PEF (18 L/s) and PIF (17 L/s) during hyperpnoea in this study were lower than results obtained following exercise (80 L/s) [18] or lobeline administration (40 L/s) [19].

During eupnoea in the current study, all horses demonstrated variable breathing patterns within the same day and on a breath to breath basis. Unsurprisingly, given that breaths were selected for analysis partly based on uniformity, the within day variation was much less than that observed between days. Some variability in tidal volumes and respiratory frequency was expected during eupnoea, as horses have significant respiratory reserve, and ventilation is subject to rapid change in response to excitement, fear and other emotional states. Additionally, horses regularly take single large breaths (sighs), which serve to redistribute ventilation within pulmonary fields [37]. Carbon dioxide-induced hyperpnoea attenuated both the within day and between day variability observed in the current study. Rebreathing was expected to reduce variability in respiratory patterns as biphasic breathing is eliminated if respiratory muscles are activated before peak passive flow is obtained [32], as occurs during increased respiratory effort for physiological or pathological reasons. The use of capnography to standardise the degree of rebreathing in this study may have further served to reduce variation in measures obtained during hyperpnoea.

Values obtained in this study for most absolute and relative respiratory indices demonstrated adequate reproducibility during eupnoeic respiration and rebreathing. Time invested acclimatising horses to the test environment and spirometry procedures likely improved the reliability of the findings of the current study, and research methodology based on selection of three consecutive breaths, representative of the available spirometry trace and based on carefully defined inclusion criteria, was also designed to optimise repeatability and precision of PFT procedures. Previous respiratory studies have utilised analysis of three breaths from each epoch [18, 37], however, analysis of a greater number of breaths might increase the accuracy of derived measures of respiratory function [23], and further analyses may be indicated to determine the optimal number of breaths for analysis. Coefficients of variation in the current study were similar to, or less than, analogous results from previous studies [11, 34], and within acceptable limits for PFT [12]. However evaluation of individual variation, mean differences and 95% confidence intervals from repeated measures analysis suggested that the technique might be better suited to repeated studies in the same individual, rather than to the establishment of absolute reference ranges and cross-sectional studies. Results from determination of ICC demonstrated that measures of expiratory time (absolute Tpef, Tpef/Te, Te/Tt and Te/Ti) had the greatest agreement which, in this context, suggests these measures were the most repeatable for individual animals. Similarly, based on ICC results, relative flow-time indices derived during eupnoeic respiration demonstrated slightly better agreement than values obtained during rebreathing.

The time at which PEF occurred within each breath was highly variable between horses in the current study. Unlike humans, dogs and cats, the mechanical equilibrium of respiration for horses is at the midpoint of tidal volume, as opposed to the end of expiration, resulting in active and passive components of both inspiration and expiration [32]. Consequently, horses may exhibit monophasic or biphasic respiratory patterns during eupnoeic breathing, with consequent variation in the position of peak inspiratory and expiratory flows between breaths. In the current study all horses demonstrated biphasic expiration during eupnoea; however inspiration was monophasic, biphasic or both. Study horses exhibited PIF and PEF variably in early, middle and late phases of respiration in apparent random distribution. Although such variability is well documented [17] and previous studies have differed in their conclusions regarding normal resting respiratory patterns in horses [20, 32, 38], it has been suggested that normal horses typically have PEF early in expiration and PIF late in inspiration [25, 32, 33]. Expiratory flows have greater sensitivity and accuracy for the diagnosis of obstructive airway disease in human patients and horses [4], and both human and equine studies have demonstrated that PEF occurs earlier in expiration in individuals with airway obstruction [22, 39, 40].

During hyperpnoea, expiration remained biphasic, whilst inspiration tended to become monophasic in the current study, in agreement with previous findings during lobeline-induced hyperpnoea [16]. Hyperpnoea induced by exercise typically results in loss of both biphasic inspiration and expiration as consequence of earlier and more intense respiratory muscle contraction and locomotion coupling [32, 38]. The current study found both PIF and PEF occurred close to the start of inspiration and expiration during rebreathing; PEF was higher and increased more than PIF, as has been previously reported [16].

There was no apparent variation attributable to day of testing in relative flow-time indices derived from spirometry traces in the current study. Variability was least for relative flow-time values obtained between peak and zero inspiratory flow (ipz) and between zero and peak expiratory flow (ezp), suggesting that these measurements might be the most suitable for experimental studies. Between day variability was reduced during hyperpnoea for all derived flow-time values, and higher relative flows were obtained earlier within each phase of the breathing cycle than was evident in eupneoic breathing. Consistent with the variable flow characteristics observed, significant individual variation was identified for most relative flow-time variables during both eupnoeic respiration and rebreathing. As was observed in the current study, relative flow-time values after PEF (epz) had the greatest variation in adult human subjects [41]. In horses in the current study, expiratory flow after PEF typically had a biphasic pattern (as PEF occurs early in expiration), therefore the values of epz25%, epz50% and epz75% may be of similar magnitude and not accurately represent flow limitation (Fig. 2). As horses with airway obstruction have reduced maximal flows in final stages of expiration [11], future experiments might focus on calculations at relative flow-time values later in the expiratory period (eg. epz 75%, 80%, 85%, 90% and 95%). Despite considerable individual horse variation, relative flow-time values derived during expiration in the current study showed the greatest agreement, as indicated by high ICC values. Again this is similar to studies on tidal breathing in adult patients [41], where evaluation of expiratory flows after PEF was useful in identifying obstructive respiratory conditions. Further studies are required to determine whether these relative flow-time indices are useful for characterisation of obstructive lower airway disease in horses. Although the current study was not intended to evaluate the diagnostic utility of breath by breath spirometry or relative flow-time indices for the diagnosis of obstructive airway conditions, and indeed used horses that were clinically healthy, endoscopy and airway cytology suggested minor changes (slight increase in tracheal mucus) were present in four of the eight horses. These changes barely fulfilled inclusion criteria for IAD [1] and had no obvious effect on indices of respiratory function in the present study.

## Conclusions

Individual horse variation observed in all relative flow-time parameters in the current study may prove a limitation in determining reference ranges for use in a diagnostic setting. However, the current study demonstrated that the mask spirometry system used was able to provide reliable (repeatable and reproducible) absolute and relative indices of respiratory function in healthy horses. Further evaluation of the technique as a tool in the diagnosis or monitoring of respiratory disease in horses is required. The reliability of the technique suggests its utility in the evaluation of repeated measures of airway function, such as would be required for bronchoprovocation tests or the assessment of the response to treatment.

## Additional files

Additional file 1: Figure S1. Representative volume (green) and flow (pink) traces obtained during spontaneous respiration showing uniform volume and flow traces which would satisfy inclusion criteria for analysis. Note biphasic expiratory and inspiratory respiration. **Table S1.** Respiratory parameters measured for each of three selected breaths. Data from each breath was evaluated to ensure inclusion criteria were met (≤10% difference in inspiratory and expiratory volumes, ≤10% difference in time to peak inspiratory and expiratory flow (Tpif, Tpef) for the three breaths). **Table S2.** Definition of relative flow-time variables calculated for each of three selected breaths. **Figure S2.** Mean difference and 95% confidence intervals between days for absolute measures (respiratory frequency, Rf; tidal volume, Vt; peak inspiratory and expiratory flows, PIF and PEF; time to PEF, Tpef) determined during pulmonary function testing over three consecutive days. **Figure S3.** Mean difference and 95% confidence intervals between days for relative expiratory flow indices (ratio of time to PEF to expiratory time; Tpef/Te; ratio of time to PEF to total breath duration; Tpef/Tt) determined during pulmonary function testing over three consecutive days. **Figure S4.** Mean difference and 95% confidence intervals between days for relative flow-time indices determined during pulmonary function testing over. (PDF 525 kb)
Additional file 2: Raw data. (XLS 414 kb)

## Abbreviations

ANOVA: Analysis of variance
BAL: Bronchoalveolar lavage
CO_2_: Carbon dioxide
CV: Coefficient of variation
epz_25%_, epz_50%_, epz_75%_: 25%, 50%, 75% time from PEF to zero flow
ezp_25%_, ezp_50%_, ezp_75%_: 25%, 50%, 75% time from zero to PEF
IAD: Inflammatory airway disease
ipz_25%_, ipz_50%_, ipz_75%_: 25%, 50%, 75% time from PIF to zero flow
izp_25%_, izp_50%_, izp_75%_: 25%, 50%, 75% time from zero to PIF
MVe: Minute ventilation
PEF: Peak expiratory flow
PFT: Pulmonary function testing
PIF: Peak inspiratory flow
PMN: Polymorphonuclear cells (neutrophils)
RAO: Recurrent airway obstruction
Rf: Respiratory frequency
Te: Expiratory period
Ti: Inspiratory period
Tpef: Time to peak expiratory flow
Tpif: Time to peak inspiratory flow
Tt: Total breath period
TW: Tracheal wash
V_t_: Tidal volume
