# Supplementary material for: Reliability of breath by breath spirometry and relative flow-time indices for pulmonary function testing in horses
Source: BMC Vet Res. 2016 Nov 28;12:268. doi: 10.1186/s12917-016-0893-3 (PMC5126818; doi:10.1186/s12917-016-0893-3)
Supplement: Additional file 1: Figure S1. — Representative volume (green) and flow (pink) traces obtained during spontaneous respiration showing uniform volume and flow traces which would satisfy inclusion criteria for analysis. Note biphasic expiratory and inspiratory respiration. Table S1. Respiratory parameters measured for each of three selected breaths. Data from each breath was evaluated to ensure inclusion criteria were met (≤10% difference in inspiratory and expiratory volumes, ≤10% difference in time to peak inspiratory and expiratory flow (Tpif, Tpef) for the three breaths). Table S2. Definition of relative flow-time variables calculated for each of three selected breaths. Figure S2. Mean difference and 95% confidence intervals between days for absolute measures (respiratory frequency, Rf; tidal volume, Vt; peak inspiratory and expiratory flows, PIF and PEF; time to PEF, Tpef) determined during pulmonary function testing over three consecutive days. Figure S3. Mean difference and 95% confidence intervals between days for relative expiratory flow indices (ratio of time to PEF to expiratory time; Tpef/Te; ratio of time to PEF to total breath duration; Tpef/Tt) determined during pulmonary function testing over three consecutive days. Figure S4. Mean difference and 95% confidence intervals between days for relative flow-time indices determined during pulmonary function testing over. (PDF 525 kb) [file 12917_2016_893_MOESM1_ESM.pdf]

Online Resource 1

Figure S1: Representative volume (green) and flow (pink) traces obtained during spontaneous respiration showing uniform volume and flow traces which would satisfy inclusion criteria for analysis. Note biphasic expiratory and inspiratory respiration.

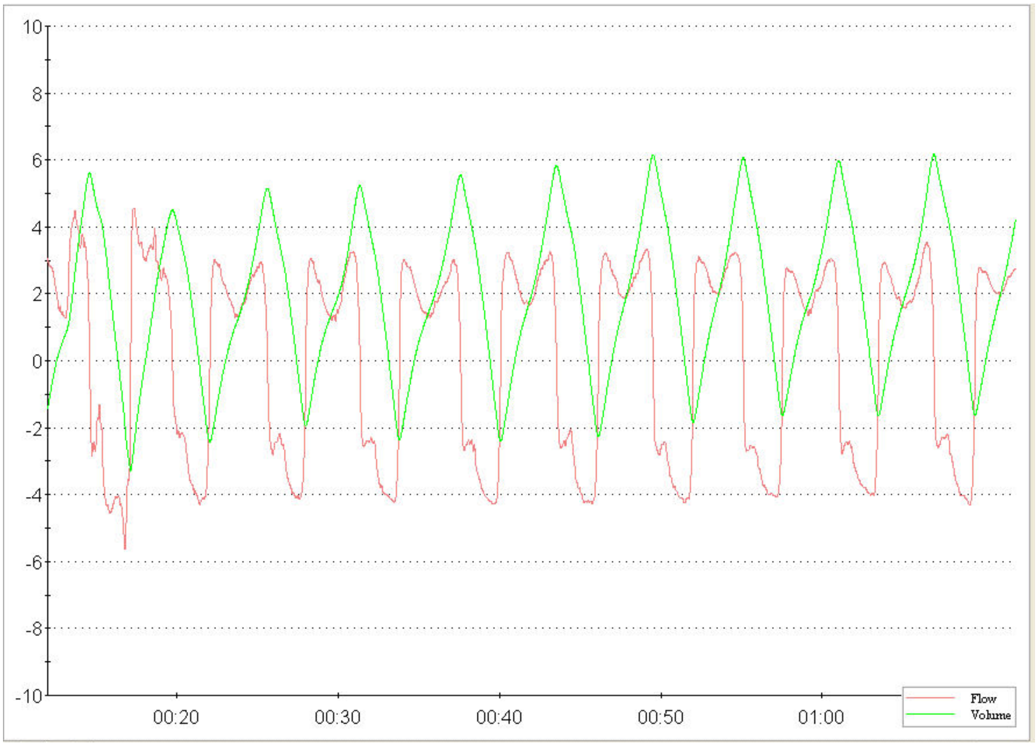

Online Resource 2

Table S1: Respiratory parameters measured for each of three selected breaths. Data from each breath was evaluated to ensure inclusion criteria were met (≤10% difference in inspiratory and expiratory volumes, ≤10% difference in time to peak inspiratory and expiratory flow (Tpif, Tpef) for the three breaths).

**Parameter**	**Abbreviation**	**Units**
Respiratory frequency	Rf	bpm
Inspired Volume	Vi	L (BTPS)
Expired Volume	Ve	L (BTPS)
Tidal Volume	Vt	L (BTPS)
Total breath period	Tt	msec
Inspiratory period	Ti	msec
Expiratory period	Te	msec
Peak inspiratory flow	PIF	L/sec (BTPS)
Peak expiratory flow	PEF	L/sec (BTPS)
Minute ventilation	MVe	L
Time to pif	Tpif	msec
Time to pef	Tpef	msec
Ratio Te to TT	Te/Tt	
Ratio of Te to Ti	Te/Ti	
Ratio of time to pef to Te	Tpef/Te	
Ratio of time to pif to Ti	Tpif/Ti	

Abbreviations: bpm – breaths per minute, L – litre, BTPS – body temperature and pressure saturated with water vapour, sec – second, msec – millisecond.

Online Resource 3

Table S2: Definition of relative flow-time variables calculated for each of three selected breaths.

**Relative flow-time variable**	**Abbreviation**
Percentage of PIF at 25%, 50% & 75% of the time from start of inspiration to PIF	izp25%<br>izp50%<br>izp75%
Percentage of PIF at 25%, 50% & 75% of the time from PIF to zero flow	ipz25%<br>ipz50%<br>ipz75%
Percentage of PEF at 25%, 50% & 75% of the time from start of expiration to PEF	ezp25%<br>ezp50%<br>ezp75%
Percentage of PEF at 25%, 50% & 75% of the time from PIF to zero flow	epz25%<br>epz50%<br>epz75%

Abbreviations: PIF, peak inspiratory flow; PEF, peak expiratory flow

Online Resource 4

Figure S2: Mean difference and 95% confidence intervals between days for absolute measures (respiratory frequency, Rf; tidal volume, Vt; peak inspiratory and expiratory flows, PIF and PEF; time to PEF, Tpef) determined during pulmonary function testing over three consecutive days.

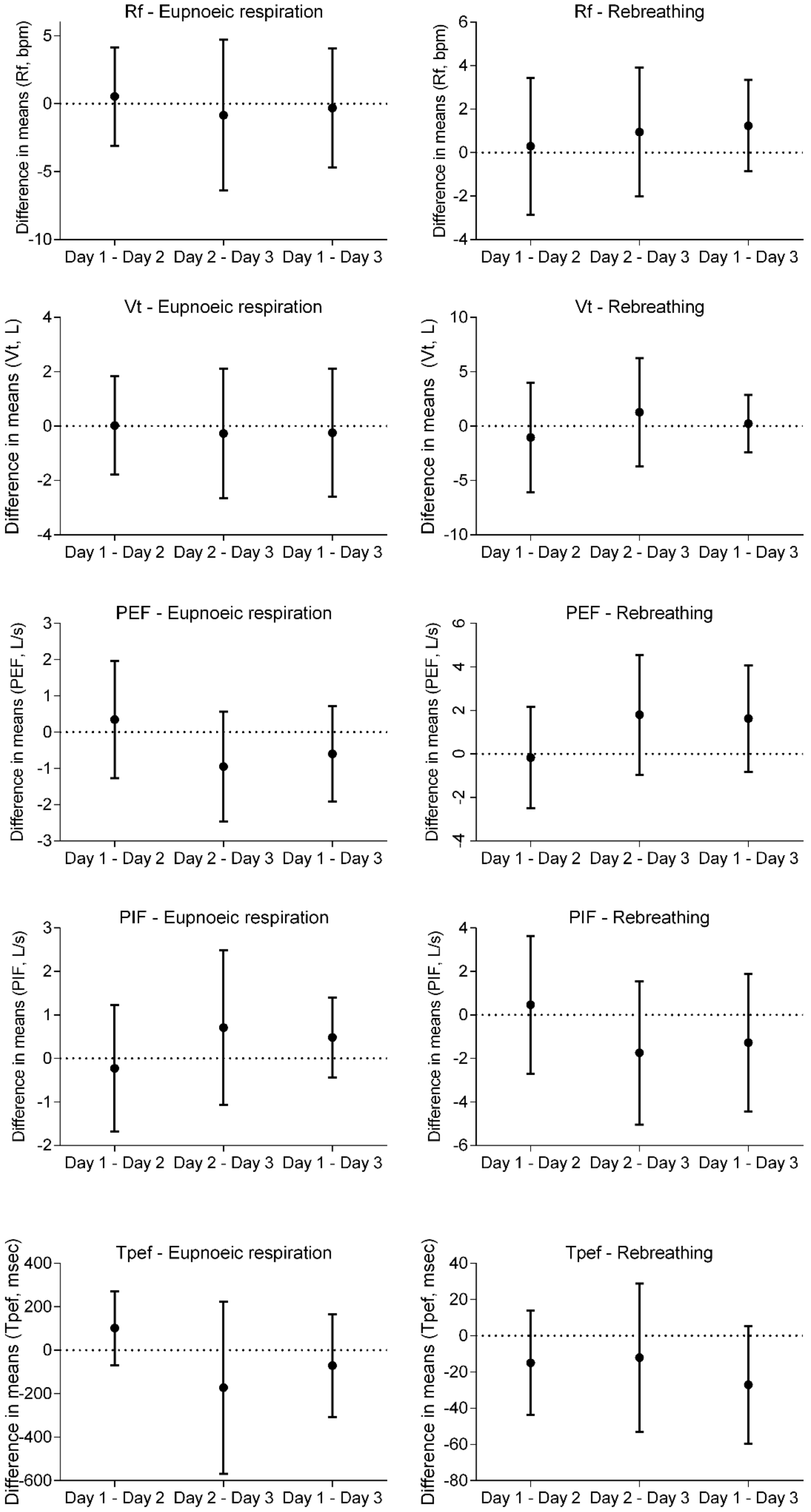

Online Resource 5

Figure S3: Mean difference and 95% confidence intervals between days for relative expiratory flow indices (ratio of time to PEF to expiratory time; Tpef/Te; ratio of time to PEF to total breath duration; Tpef/Tt) determined during pulmonary function testing over three consecutive days.

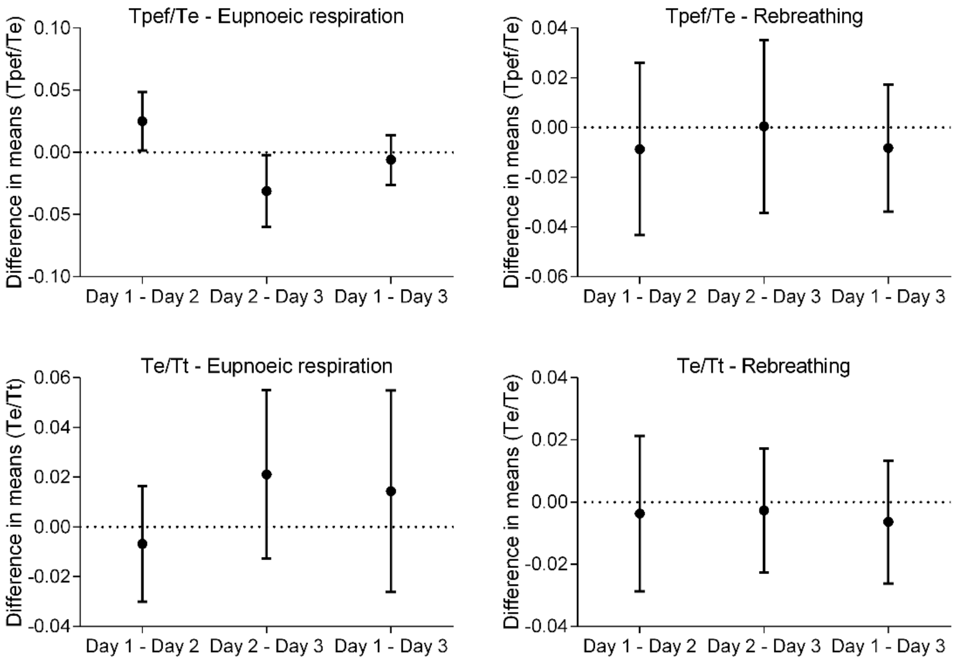

Online Resource 6

Figure S4: Mean difference and 95% confidence intervals between days for relative flow-time indices determined during pulmonary function

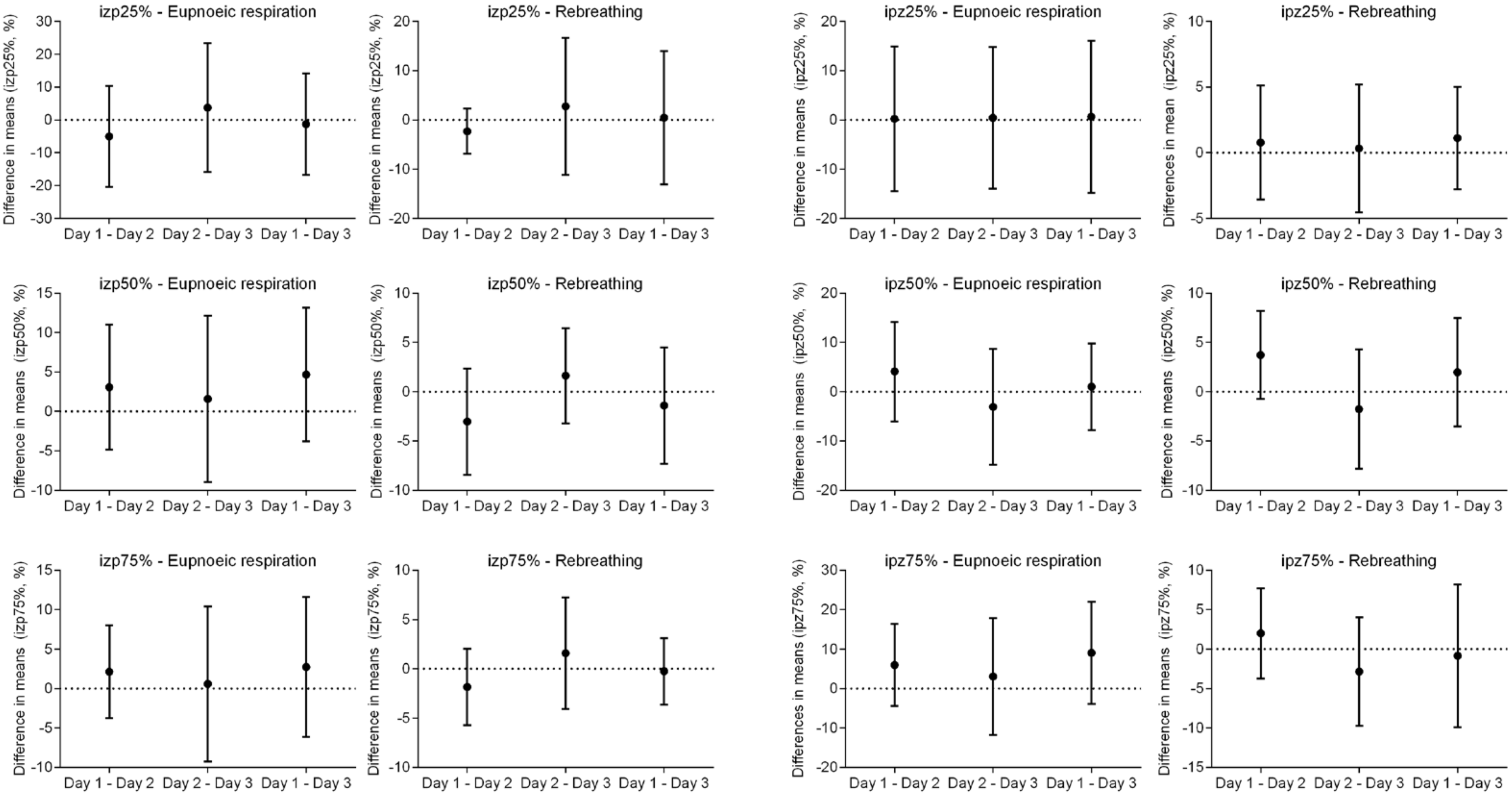

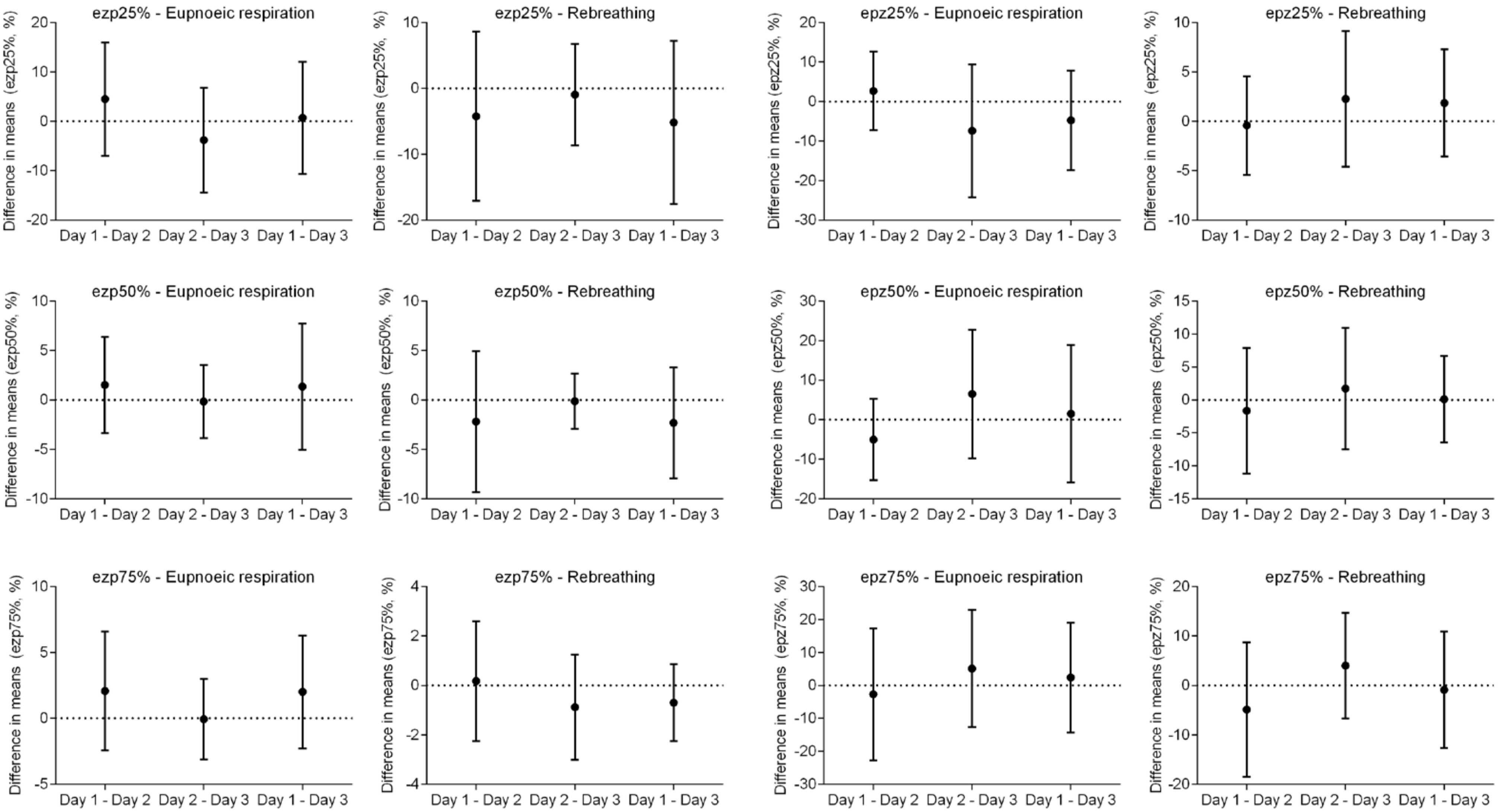
ezp25% - Eupnoeic respiration
ezp25% - Rebreathing
epz25% - Eupnoeic respiration
epz25% - Rebreathing
ezp50% - Eupnoeic respiration
ezp50% - Rebreathing
epz50% - Eupnoeic respiration
epz50% - Rebreathing
ezp75% - Eupnoeic respiration
ezp75% - Rebreathing
epz75% - Eupnoeic respiration
epz75% - Rebreathing
Difference in means (ezp25%, %)
Difference in means (epz25%, %)
Difference in means (ezp50%, %)
Difference in means (epz50%, %)
Difference in means (ezp75%, %)
Difference in means (epz75%, %)
Day 1 - Day 2
Day 2 - Day 3
Day 1 - Day 3
